# Porting Rulex Software to the Raspberry Pi for Machine Learning Applications on the Edge †

**DOI:** 10.3390/s21196526

**Published:** 2021-09-29

**Authors:** Ali Walid Daher, Ali Rizik, Marco Muselli, Hussein Chible, Daniele D. Caviglia

**Affiliations:** 1 COSMIC Lab, Department of Electrical, Electronic and Telecommunications Engineering and Naval Architecture (DITEN), University of Genoa, 16145 Genoa, Italy; ali.daher@edu.unige.it (A.W.D.); ali.rizik@edu.unige.it (A.R.); 2 MECRL Laboratory, Ph.D. School for Sciences and Technology, Lebanese University, Beirut 6573/14, Lebanon; hchible@ul.edu.lb; 3 Consiglio Nazionale delle Ricerche, Institute of Electronics Computer and Telecommunication Engineering (IEIIT), 16149 Genoa, Italy; marco.muselli@ieiit.cnr.it; 4 Rulex Innovation Labs, Rulex Inc., 16122 Genoa, Italy

**Keywords:** edge computing, internet of things, machine learning, image classification, pre-processing

## Abstract

Edge Computing enables to perform measurement and cognitive decisions outside a central server by performing data storage, manipulation, and processing on the Internet of Things (IoT) node. Also, Artificial Intelligence (AI) and Machine Learning applications have become a rudimentary procedure in virtually every industrial or preliminary system. Consequently, the Raspberry Pi is adopted, which is a low-cost computing platform that is profitably applied in the field of IoT. As for the software part, among the plethora of Machine Learning (ML) paradigms reported in the literature, we identified Rulex, as a good ML platform, suitable to be implemented on the Raspberry Pi. In this paper, we present the porting of the Rulex ML platform on the board to perform ML forecasts in an IoT setup. Specifically, we explain the porting Rulex’s libraries on Windows 32 Bits, Ubuntu 64 Bits, and Raspbian 32 Bits. Therefore, with the aim of carrying out an in-depth verification of the application possibilities, we propose to perform forecasts on five unrelated datasets from five different applications, having varying sizes in terms of the number of records, skewness, and dimensionality. These include a small Urban Classification dataset, three larger datasets concerning Human Activity detection, a Biomedical dataset related to mental state, and a Vehicle Activity Recognition dataset. The overall accuracies for the forecasts performed are: 84.13%, 99.29% (for SVM), 95.47% (for SVM), and 95.27% (For KNN) respectively. Finally, an image-based gender classification dataset is employed to perform image classification on the Edge. Moreover, a novel image pre-processing Algorithm was developed that converts images into Time-series by relying on statistical contour-based detection techniques. Even though the dataset contains inconsistent and random images, in terms of subjects and settings, Rulex achieves an overall accuracy of 96.47% while competing with the literature which is dominated by forward-facing and mugshot images. Additionally, power consumption for the Raspberry Pi in a Client/Server setup was compared with an HP laptop, where the board takes more time, but consumes less energy for the same ML task.

## 1. Introduction

The Internet of Things paradigm is rapidly extending to many sectors of society because it allows to substantially improve the monitoring or control of complex and extensive processes, offering an innovative approach for multiple fields of application, such as quality of life, urban challenges, logistics, agriculture and livestock, climate change, mass production, health, energy and water production and distribution, and many more.

The huge amount of data produced by the nodes of the networks of which the Internet of Things is made up must be processed in an efficient and effective way, and ML techniques are certainly among the most suitable for this purpose. Therefore, it is straightforward that Machine Learning tools can play a key role in further expanding the scope of applications, as well as their effectiveness. In past implementations, Machine Learning forecasts have been performed on a remote server before delivering results on the IoT Computing Node to limit network traffic, Edge Computing [1,2] setups can be employed to avoid intensive cloud access and keep that data storage and processing on the IoT device as much as possible.

Our work is placed in this perspective. Notably, this paper reports an extended version of the work previously presented at ApplePies 2020 [3]. The system we report is based on the Raspberry Pi platform [4,5], which is a low-cost, low-power credit-card-sized board that is used for embedded system and general-purpose computing applications. As for Machine Learning software, we have adopted for this investigation Rulex [6], which can be found online in [7], an AI (Artificial Intelligence) environment intended for non-domain experts, and we have ported it to the Raspberry Pi platform.

Rulex was ported to three different Operating Systems, namely to Windows 32 Bits, Ubuntu 64 Bits, and on Raspbian 32 Bits which is the official Operating System (OS) of the Raspberry Pi. All external and internal dependencies have been compiled [3] and verified. Moreover, the Client/Server setup has been used to perform forecasts on the edge after debugging the software through its source code.

To explore the application possibilities of this operating environment, in addition to the Radar Classification dataset already reported in [3], three new pre-processed datasets taken from diverse domains were also implemented using Rulex on the Edge. These encompass a Human Activity Detection dataset using Smartphones, a Brainwave Mental State Classification dataset, and an Activity Recognition dataset for Dumpers in earth moving sites that are also recorded using Smartphones. We also investigated the problem of performing gender detection. To this end, a new pre-processing Algorithm for image classification was developed to convert facial images into Time-series using a contour-based approach.

The contributions presented in this paper include the porting of a high performance ML package on the Raspberry Pi in a Client/Server setup, as well as the development of a novel pre-processing Algorithm that converts images into Time-series using statistical measurements, that are directly applicable to any ML configuration.

The methodology in the paper consists of compiling all the required libraries on the target platforms. The correct version of the libraries should be chosen where all libraries should be compatible with their predecessor since libraries may be built on top of each other. Also, in the linking process, the code needs to be changed to satisfy all the target platforms (Windows 64-bits, Windows 32-bits, Raspbian 32-bits, and Ubuntu 64-bits). Finally, the fully featured Rulex software package can be run on open-source hardware. Additionally, the pre-processing contribution and the ML tests on the system using multiple datasets and, in a Client/Server arrangement demonstrate the system effectiveness.

In the rest of this paper Section 2 presents the literature review, Section 3 describes the actions taken to bring Rulex on Raspberry Pi in a Client/Server arrangement [8], Section 4 presents in detail the image pre-processing Algorithm, and reports the results of all the ML forecasts and energy consumption achieved. Finally, Section 5 draws the conclusions. 

## 2. Literature Review

### 2.1. Hardware Platform

The hardware platform adopted in this work is the multi-purpose Raspberry Pi [4,5]. It was conceived to handle application-specific tasks, as well as being used for everyday computing operations. It supports Universal Serial Bus (USB), HDMI, and SD Card connections, in addition to having standard digital Input/Output pins controlled through the on-board ARM microcontroller. 

Regarding Communication protocols, the Raspberry Pi orts Local Area Network (LAN) and Wi-Fi connectivity. Furthermore, the Raspberry Pi’s digital pins can be used for interfacing with a wide variety of peripherals, for applications ranging from motor control, LCD Display functions, and may more and can be interfaced with compatible smart sensor modules.

### 2.2. Machine Learning Platform

The AI suite named Rulex (an acronym for Rule Extraction) has been created specifically for the management, the visualization, and the analysis of data: it consists in an integrated visual platform which allows to perform any operation in a simple and direct way, freeing the user from the necessity of knowing implementation details about memorization and execution [4]. It actually implements many Machine Learning Algorithms (both supervised and unsupervised) such as Logic Learning machine (LLM) [9], Neural Networks, Binary Trees, K-Nearest Neighbor (KNN), and others through an easy-to-use Graphical User Interface (GUI). Specifically, the Logic Learning Machine (LLM) is a method of supervised analysis based on an efficient implementation of the Switching Neural Network [10] and Monotone Boolean Reconstruction [11] through the Shadow Clustering technique [12] and is able to extract intelligible rules from data.

Rulex, through its GUI, also allows you to choose and apply other standard ML Algorithms to perform predictions. Through the interface, it is also possible to manipulate and filter data before applying forecasts using the same software package. Rulex [4] is natively supported on Windows 64 Bits PC’s, where it operates in a standalone setup, storing its workflows on a local database. 

### 2.3. Machine Learning and IoT Systems

As mentioned in the previous section, IoT is being applied to many applications and systems. For example, authors in [13] propose and edge computing framework for collaboration among nodes with the aim to improve resources management and achieve optimal offloading directed towards healthcare systems. Also, energy consumption on the edge and the used of ML to improve its performance is addressed in [14,15,16], since energy consumption is essential during ML forecasts due the limited power supplies available for light-weight IoT devices. Furthermore, authors in [17] apply ML algorithms for an indoor classification applications which uses features collected from radio frequency measurements. Also, ML forecasts are applied on time-series in [18] to predict failure on a slitting machine by relying on data collected by IoT sensors. Additionally, ML is used to secure IoT networks in [19,20] while improving systems security using Neural Networks.

Therefore, due to the vast scope of application of ML on the edge including its possible use in energy, healthcare, security, and resource allocation, the main purpose of this paper was to deploy a fully featured ML package on the edge to expand its services to the field of IoT. 

## 3. Porting Techniques and Tools

The fundamental task of this project is the porting of the Rulex from 64 bit to 32-bit platforms. In the case of Windows, the Visual Studio environment to accomplish this task, however, in the case of Raspbian 32-bits and Ubuntu 64-bits, CMake [21,22] was employed to compile the external and internal dependencies and port Rulex.

**Figure 1:**
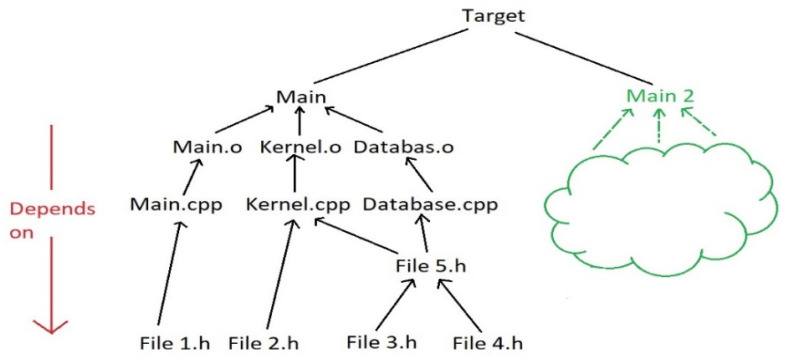
Dependencies file structure [3].

In Figure 1, a tree-based file structure is shown, which presents a set of libraries or dependencies that exist in a porting process. The header files contain functions that are called in Cpp files. These header files may depend on other header files, however, Cpp files cannot call a function unless the corresponding header file is included. The Cpp files generate their output files which produce an overall output binary file. A binary file contains a compiled or encrypted version of the functions found in a header file. Consequently, binary files could depend on other binary files, where in general, the final target is an executable.

Rulex on the Raspberry Pi can operate in Standalone mode or Client/Server mode. In the latter case, a Windows 32-bit system is used as a client, where the Rulex GUI is running, and the Raspberry Pi functions as a server or ML engine. An SSH connection [23] is used in case the connection is over a public or private network. After connecting to the Rulex Engine on the Raspberry Pi, remote development was used to debug the code so it can operate on both Windows 32 Bits as well as Linux 32 Bit/ 64 Bit such that it runs without any manual modification. 

In the process of testing, the software runs showed that some of the original C/C++ variables from the Windows 64-bit version are not compatible with 32 Bit systems. Therefore, it was necessary to make the source redundant concerning this issue. So, the flow of the code was diverted to a path or snippet specific to the running OS, which is implemented using Macros. For example, a Macros such as _WIN32 was used to detect a Windows 32-bits OS and _WIN64 for detecting Windows 64-bits. Also, to detect ARM, the __arm__ Macro was executed. 

Rulex GUI source code was debugged through the SSH connection where a PC acts as the Client, and the Raspberry Pi forming the ML Server. In the Client/Server setup, a Docker-based PostgreSQL container [24] is placed as the common storage point between the Client and server nodes. 

## 4. Forecast Results

To verify the effectiveness of the proposed solution, after porting Rulex to Windows 32 Bits as a Client, and Raspberry Pi as an application server, we tested Rulex on multiple datasets from five diverse applications having a different number of samples and with varying dimensions. Additionally, we’ve tested the accuracy of the Image-to-Timeseries pre-processing Algorithm on a gender classification dataset.

### 4.1. Radar Classification

The Urban Classification dataset was recorded by a short-range 24 GHz radar, based on the Infineon BGT24MTR11 RF transceiver [25], and particularly, the Distance2Go development kit by Infineon [26].

For ML forecasts, four classes were considered: One for *Humans* and three vehicle classes. Namely, Car, Truck, and Motorcycle where the dataset contains 120 records. In [27], a multiclass tree-based classification technique was implemented to improve the prediction accuracy using this dataset.

The radar data has been recorded using another separate system dedicated to feature extraction. This standalone system is composed of three parts. Firstly, A 24 GHz radar, a second Raspberry PI 3B+, and a PC running MATLAB. This second Raspberry PI was employed to connect the MATLAB station to the radar board. The Raspberry PI collects the data from the radar, and then it sends it to MATLAB running on a PC where feature extraction is applied [28,29].

Below is a list of the features extracted using the radar measurements:

1. R: The spread in the range-FFT spectrum caused by the target.
2. R_1_: Variance of the range-FFT spectrum.
3. R_2_: Standard deviation of the range-FFT spectrum.
4. R_3_: Average of the range-FFT spectrum.
5. V: The spread in the Doppler-FFT spectrum caused by the target movement.
6. V_1_: Variance of the Doppler-FFT spectrum.
7. V_2_: Standard deviation of the Doppler-FFT spectrum.
8. V_3_: Average of the Doppler-FFT spectrum.
9. RCS: Radar Cross-Section, that gives a measure for the reflectivity of the target.
10. V_est_: The estimated speed of the target.

**Figure 2:**
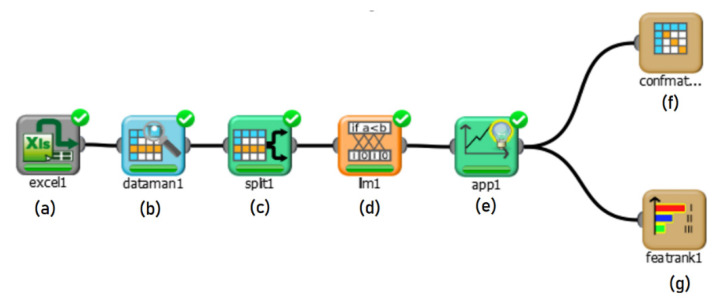
Rulex processing blocks: consisting of (**a**) excel1 which imports data, (**b**) dataman1 for viewing and data mining, (**c**) split1 for splitting dataset, (**d**) lm1 which performs the machine learning algorithm, (**e**) app1 to apply the model, and finally (**f**) confmatrix1 and (**g**) featrank1 which visually display results [3].

After extracting features using the second system, the first Client/Server setup which consists of Rulex running on a Raspberry PI is used for ML predictions. An example of a workflow in Rulex GUI running on the Raspberry is presented in Figure 2, where there are data processing blocks followed by an LLM, and finally a confusion matrix. Moreover, a block that splits data for training and testing is shown, where the ratio is 65% for training and 35% for testing.

**Figure 3:**
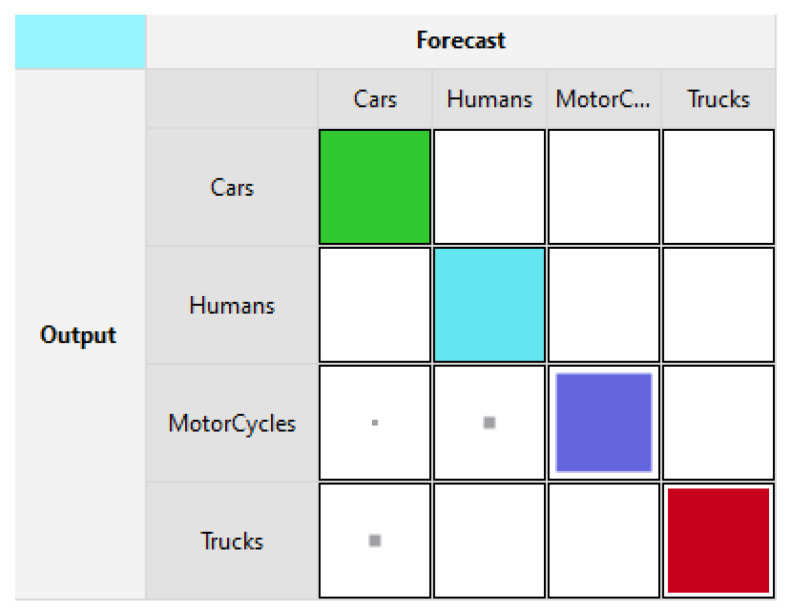
Radar training forecast [3].

**Figure 4:**
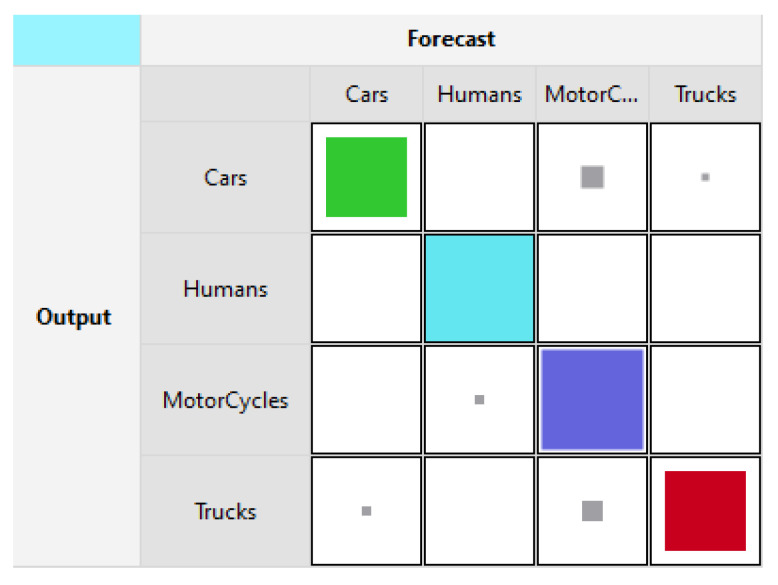
Radar testing forecast [3].

Figure 3 and Figure 4 show the training and testing accuracies using LLM. In Figure 3, Cars and Humans are classified with a rate of 100%. As for Motorcycles and Trucks, they are 84.2% and 89.5% respectively. Figure 4 shows the testing accuracies where Cars are detected with a rate of 73.3%, Humans at 100%, Trucks at 72.7%, and Motorcycles were recognized with a rate of 90.9%.

### 4.2. Human Activity Detection Using Smartphones

The Activity dataset from [30] consists of features that have been recorded using the Accelerometer and Gyroscope that are included in a Smartphone. The dataset is pre-processed such that it can be directly applied in Rulex since the features are composed of basic statistical operations based on the measurements. These consist of the mean, STD, variance, and others. The activity performed by a subject is divided into six classes: Laying, Standing, Sitting, Walking, Walking Upstairs, and Walking Downstairs.

**Figure 5:**
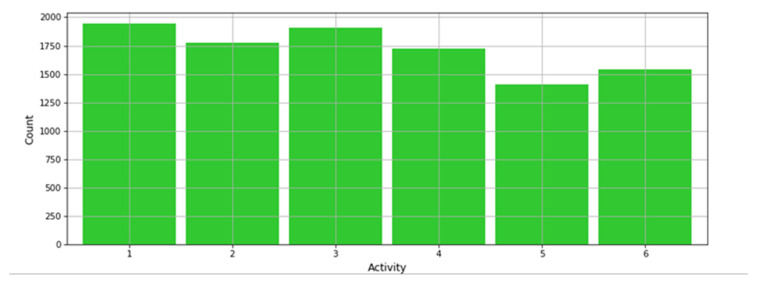
Low class skewness for the Human Activity Detection dataset.

A subset of the original dataset was used which is reduced to 7530 samples and 560 features, excluding the subject field which identifies the person or test subject specifically. When this field is included, the accuracy is increased considerably, however, it does not consider that the Smartphone can be carried by different people. This has been done to test the robustness of the ML forecasts and investigate the effectiveness of the present experiment. Furthermore, the dataset labels are distributed equally as shown in the histogram from Figure 5.

The ML forecasts for the Smartphone Activity Detection dataset are provided by Table 1, wherein the Rulex Software, three basic ML Algorithms are implemented: LLM, K-Nearest-Neighbor (KNN), and SVM. All tests have been applied in a Client/Server arrangement with the Rulex Engine running on the Raspberry Pi. As shown in Table 1, high testing accuracy was reached in every forecast, where most notably, in the case where SVM is applied, a near-perfect accuracy is achieved.

### 4.3. Brainwave Mental State Classification

**Figure 6:**
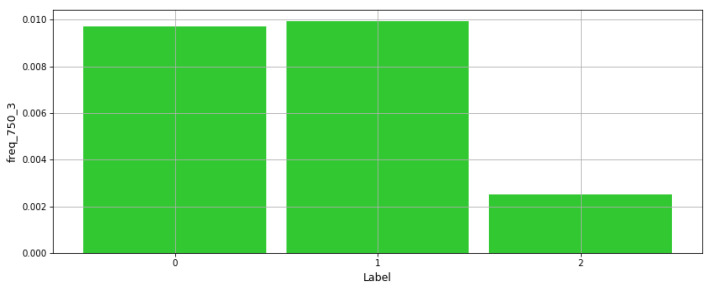
Distribution of frequency-based feature values vs. class labels.

**Figure 7:**
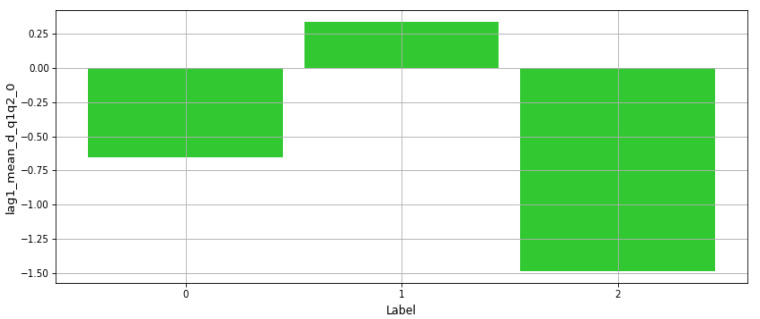
Distribution of mean-based feature value vs. class labels.

In [31], Electroencephalogram (EEG) recordings were used to predict the mental state of a subject through ML techniques. In this paper, the features from the concerned dataset are used in multiple forecasts to classify the mental into three given classes: Relaxed, Neural, and Concentrating states. Multiple Algorithms have been implemented consisting of the three basic ML Algorithms: SVM, Neural Networks and KNN, where SVM achieved the best performance. Figure 6 presents a plot of one frequency-based feature value vs. class labels, where this feature exhibits a lower value for one of the labels. Additionally, Figure 7 presents the same plot for another mean-based feature from the dataset, where results show that this feature has varying levels for each of the three classes. This illustrates the influence on every feature on each of the labels.

In this paper, we applied forecasts using Rulex running on the Raspberry Pi and in a Client/Server setup. Regarding the dataset, a total of 2480 samples and 988 features were employed for both training and testing in a 70/30 split. The accuracies for each forecast are presented in Table 2.

As shown, LLM, KNN, SVM have high testing accuracies thus demonstrating the robustness of the experiment, where the most accurate forecasts were achieved using SVM with an overall accuracy of 95.47%.

In addition to applying forecasts on the edge for this dataset, we’ve studied the power consumption of the Raspberry Pi board by recording the elapsed time and estimating the power consumption, while comparing it with the energy consumed by an HP Laptop. The results can be viewed in Table 3 and Table 4, whereas shown for LLM and SVM, the Raspberry Pi achieves more time but less energy to perform the same task.

### 4.4. Vehicle Activity Recognition

**Figure 8:**
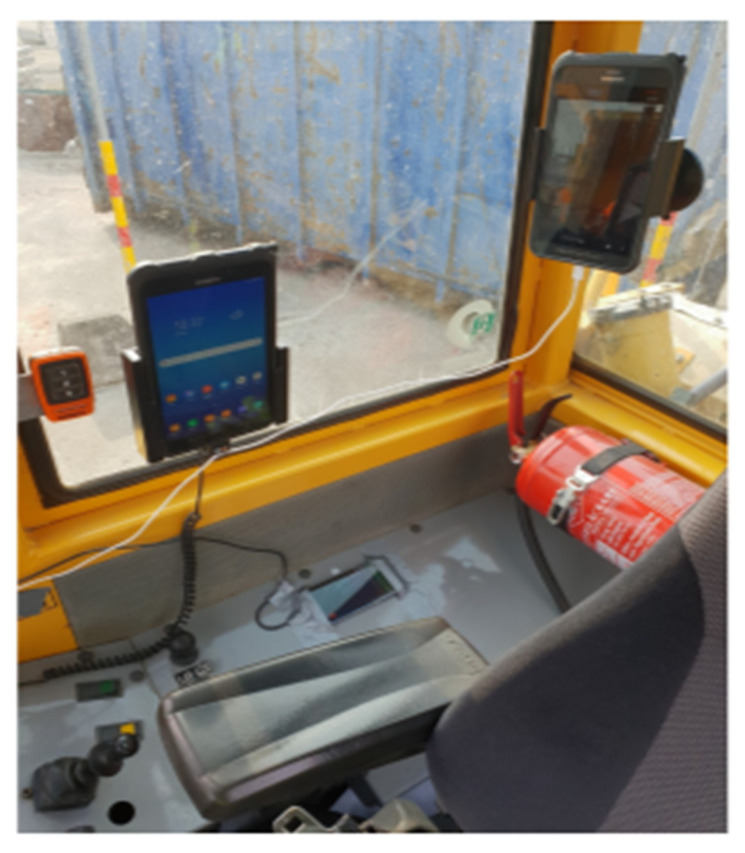
Taking sensor measurements using Smartphones to classify the state of a Dumper in an earth-moving site [32].

A dataset for Activity Recognition of Dumpers in earth moving sites is presented in [32]. It utilizes data taken from Smartphone sensors such as Gyroscopes and Accelerometers to record signals (while the Dumper is working) for feature extraction. An illustration of the data collection phase is presented in Figure 8, with the Smartphone installed inside the Dumper for taking measurements.

**Figure 9:**
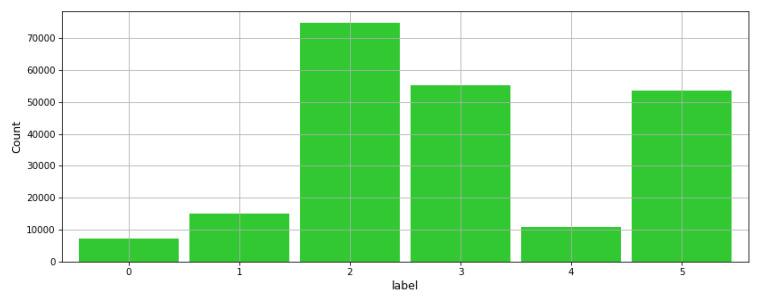
High class skewness for the Vehicle Activity Recognition dataset.

**Figure 10:**
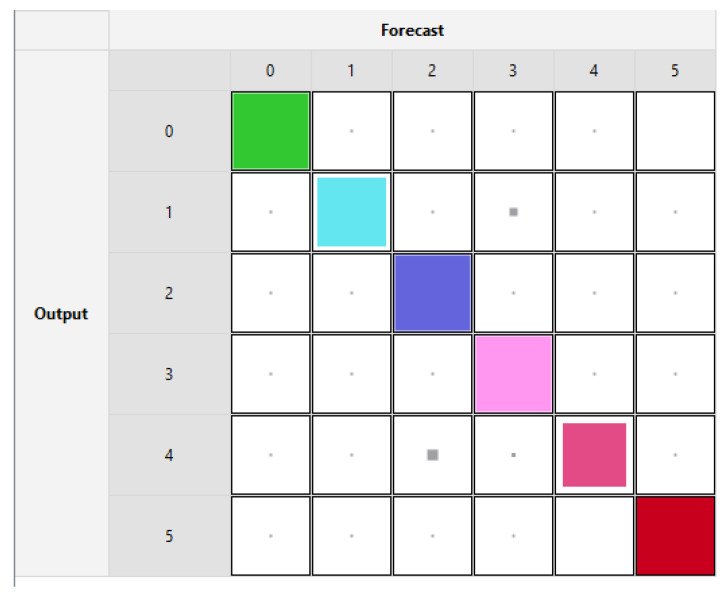
Testing Accuracy for Vehicle Activity Recognition using KNN.

The pre-processed dataset was used to classify the state of the vehicle, whether a Dumper is in one of six states, namely: idle, driving, loading, dumping, engine-off, and unknown. Figure 9 illustrates the dispersal of the labels in the dataset where there is clear skewness in the class distribution. A subset of around 216,000 samples having just 8 features were used as a data source to apply ML training and testing with a 70/30 split. Forecasts were applied through Rulex running on the Raspberry Pi in a Client/Server arrangement using the KNN Algorithm. The testing accuracy in confusion matrix format is presented in Figure 10, where the overall forecast accuracy using KNN on the Edge is 95.27%. Furthermore, with regards to power consumption, as employed in the previous dataset, the time elapsed to perform an ML task is recorded to estimate the consumed energy. In the case of the HP laptop which consumes around 70 W, as shown in Table 3, the time taken for KNN is 11.82 min and the energy is 49.63 KJ. As for the Raspberry Pi, where the power is 4 W, the time taken is 142.4 min and the energy consumed is 34.18 KJ.

### 4.5. Gender Classification

Gender classification can be useful for performing studies if implemented in an automated manner. In [33], gender detection using names, countries, and facial images is discussed where authors found that the accuracy is strongly dependent on the country, meaning that ethnicity can play a role in prediction accuracy.

Classification of Gender that is based on forward-facing images is reported in [34], where a minimum error rate of 2.85% is achieved. A Classifier implemented using Support Vector Machines (SVM) that detects gender with a minimum error of 3.4% is presented [35], where mug-shot images are used for training and testing. 

In practice, when implementing gender classification, facial images can be misaligned in contrast to some forward-facing datasets that are seldom used in the literature. Therefore, authors in [36] apply the dropout technique and SVM to tackle this issue and classify age and gender under “in the wild ” conditions. 

In this paper, we’ve implemented a novel Algorithm that converts facial images from various ethnicities, poses, zoom levels, and ages into Time-series that are generated using a radial scanning-based approach [37]. Moreover, a Sobel filter [38] is used to detect the edges of an image and with the 360° radial scan, the distance from the center (which is also determined in Algorithm 1 till every visibly detected pixel is calculated. statistical functions of this distance for every degree in the scan are computed, before generating a Time-series for every function. Finally, the Time-series are grouped to form a dataset that is used in an ML forecast.
**Algorithm 1:** Extracting center points from sobel filter output**Input:***image dataset***Output:***arrays sx, sy, centerx, centery*1.**for***i in all images****do:***2.
*sobel* = *sobel(current image)*3.
*sx [i], sy [i]* = *shape of sobel*4.
***for****positions of all horizontal points:*5.

*sumx* = *sum of points where (pixel > threshold)*
6.

***if****sumx > 0****then:***7.


*meanx = meanx + position*8.


*countx++*9.

***end if***10.
***end for***11
*centerx [i] = meanx/countx*12.
***for****positions of all vertical points:*13.

*sumy = sum of points where (pixel > threshold)*14.

***if****sumy > 0****then***:15.


*meany = meany + position*16.


*county++*17.

***end if***18.
***end for***10.
*centery [i] = meany/county*20.***end for***21.***Return****sx, sy, centerx, centery*

#### 4.5.1. Image-to-Time Series Using Statistical Radial Scanning and Sobel Filters

Image Classification usually requires specialized ML Algorithms such as Convolutional Neural Networks (CNN) which are used for applications like object detection and face recognition [39]. Most applications using CNN’s are dependent on the color of an image and its distribution. Although, some applications may not be so much reliant on color but rather the shape or contour of an image. 

With a different approach, we developed a novel Algorithm aimed to perform feature extraction for these types of applications. Specifically, it has been designed to reduce the execution time, requiring less processing power compared to CNN which demands sufficient resources [40]. Furthermore, using this approach it is possible to apply the created Time-series to any ML Algorithm thus increasing the number of possible setups.

Firstly, as shown in the pseudo-code reported in Algorithm 1, a Sobel filter extracts the edges of each input image before determining the center points of Sobel output. Initially, for every horizontal level, the sum of pixels having brightness greater than a predefined threshold is computed. Then, for each horizontal level, the mean (meanx) of the positions where the threshold was exceeded is computed. The value meanx corresponds to centrex, which is the center of filtered image. The same steps are followed to extract centery which is the vertical center of the same image. 

In summary, Algorithm 1 calculates the vertical and horizontal means for every level to determine the image center’s coordinates. Subsequently, As shown in Algorithm 2, radial scanning is performed on the Sobel filter’s output. In Algorithm 2, the outer loop is used to scan the entire 360° around the center with coordinates (centerx, centery), and In case a pixel is brighter than a Threshold, the distance from the center is calculated, where statistical functions (mean, median, STD, variance, maximum, and minimum) are computed for the distances of every angle. Therefore, multiple corresponding Time-series are generated, however, with different lengths for each record. Finally, these Time-series are interpolated (To make each set of Time-series equal, and to have the same number of features for every record) and grouped in one file to form a dataset applicable for ML predictions. The pseudo-code from Algorithms 1 and 2 has been implemented in the Python programming language to perform the Image-to-Timeseries pre-processing before performing ML forecasts on the Edge using Rulex.
**Algorithm 2:** Statistical radial scanning of images**Input:***image dataset and arrays sx, sy, centerx, cenetry***Output:***pre-processed dataset*1.**for***i in all images****do:***2.
***for**every angle y in sobel (0–90°)**do:***3.

***if**2nd iteration in loop or more****then:***4.


*rotate sobel by 90°*5.

***end if***6.

***if**y < 45°***then:**7.


*ratio = y/45*8.

***end if***9.

***if****y > 45°**then:***10.


*ratio = 45/(90.01 − y)*11.

***end if***12.

***for****k = centery[i]; k < sy[i]; k = k + 1****do:***13.


***for** j = centerx[i]; j < sx[i]; j = j + 1 **do:***14.



**if** k > (j * ratio) * 0.9 **and** k < (j * ratio) * 1.1 **then:**15.




***if** sobel(j, k) > treshsold **then:***16.





*append*$\sqrt{{\left(j-centerx\left[i\right]\right)}^{2}+{\left(k-centery\left[i\right]\right)}^{2}}$ to D17.




***end if***18.



***end if***19.


***end for***20.

***end for***21.

*“Add mean, median, STD, variance, maximum, and minimum of D to a Times-series”*22.
***end for***23.***end for***24.***task:** Interpolate all six Time-series such that every unit (record) is expanded till the max length before grouping them into a dataset to apply ML forecasts.*

#### 4.5.2. Gender Classification Experimental Results

**Figure 11:**
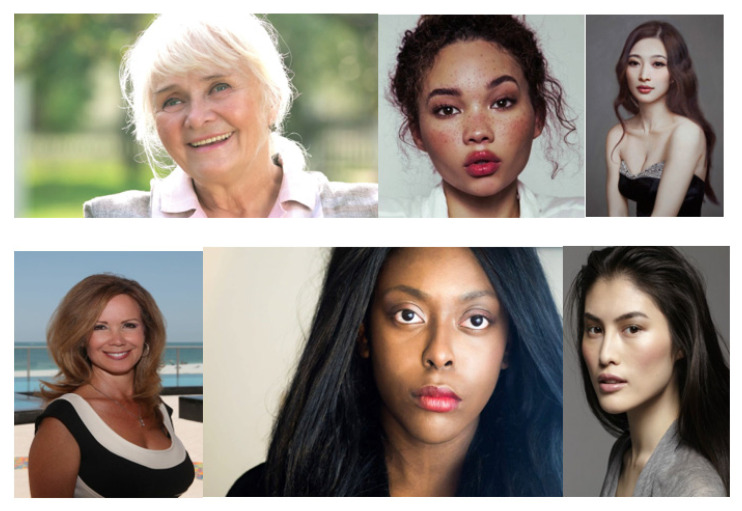
Six random images of women with varying ages, ethnicities, image background, dimensions, poses, and zoom level taken from the dataset found in [41].

To test the novel image pre-processing Algorithm described in the previous section, we considered a gender image classification dataset taken from [41]. It consists of two classes: Male and Female. The images are random where no specific angle is adopted as a reference, but rather the images of both classes have been taken from various inconsistent conditions in terms of angle, dimensions, zoom level, and background (cfr. Figure 11).

Also, some of the photos contain part of the body of the subject. Furthermore, the people in the photos have very wide ranges of age, ranging from youthful to elderly, as well as belonging to different ethnicities while taking different poses. This is demonstrated in Figure 11, where an example of six random female subjects is shown.

The dataset consists of 2700 female photos and 2720 male photos. These photos were pre-processed using the novel algorithm developed in this paper to generate the corresponding dataset that consists of sets of Time-series.

The generated dataset has been used for ML forecasts using Rulex on the Raspberry Pi. KNN and SVM were used to perform training and testing with an 80/20 split, where the results for the forecasts performed on the Edge are presented in Table 5.

In [34], the images used for gender classification were composed of people belonging to a single ethnicity using that are all forward-facing to the camera. A minimum error rate of 2.84% (accuracy of 97.16%) was achieved using SVM. Another case where SVM is used is reported in [35], where low-resolution forward-facing images are used to reach an error rate of 3.4% (96.6% accuracy). However, mug-shots or forward-facing photos may not be available in real-time practical situations, therefore, authors in [36] address this issue and attempt to determine the age and gender of random inconsistent images where the highest gender classification accuracy is 88.4%, (which is significantly lower than the publications where forward-facing photos were used) taken as the best from numerous forecasts. 

As shown in Table 5, the Image-to-Timeseries conversion Algorithm is capable of pre-processing random images (Where color is not an issue) and can be applied to various ML Algorithms to achieve high gender detection accuracy. Consequently, it can compete with previously published techniques that rely on impractical (consistent) mug-shot images for classification.

## 5. Conclusions

In this paper, we refer to the porting of Rulex, a machine learning software that natively runs on Windows 64 Bits, on the Raspberry Pi, for Edge Computing applications. We also reported the results obtained in different application domains, namely with five unrelated datasets, which we used to test the performance of our implementation in real IoT environments.

The main result obtained is that Rulex now operates in a Client/Server setup with the interface being operated on a PC as a Windows 32 Bits application, with the machine learning Algorithms being run on the Raspberry Pi ARM 32-Bit microcontroller.

As regards the performance obtainable through this implementation, first we considered a dataset related to the classification of pedestrians and vehicles using a high-frequency radar: An ML workflow was implemented on our platform and good results were achieved. However, as such dataset is relatively small, we considered four additional datasets from diverse application fields. Three pre-processed datasets having a larger number of samples and with low and high dimensionality and varying skewness were adopted. The pre-processed datasets include a Human activity Recognition dataset using Smartphones, a vehicle activity detection dataset, and an EEG Classification dataset related to mental state.

Furthermore, a novel pre-processing Algorithm was developed and implemented in Python, that converts images into Time-series to pre-process a gender detection dataset that contains inconsistent facial images in terms of the background and dimensions of the image itself and the age, ethnicity, and pose of the subject. Also, the accuracy achieved for gender classification is considerably competitive with the literature, where the competition is dominated by mug-shot and forward-facing images. Moreover, in every forecast, the training and testing were performed using Rulex on the Edge, where in general, each experiment achieved high classification accuracy through the Client/Server interface. Also, the power consumption was investigated by comparing the Raspberry Pi as an edge computing node with an HP laptop, where for the same ML Algorithm and dataset, the Raspberry Pi consumes less energy.

## Figures and Tables

**Table 1:** Forecast accuracy for the Smartphone Activity Detection application.

Human Activity Classification with Rulex			
Algorithm	LLM	KNN	SVM
Laying	100%	100%	100%
Standing	86.8%	94.4%	98.4%
Sitting	88.48%	91.86%	97.63%
Walking	94.74%	100%	100%
Walking Upstairs	89.89%	99.47%	100%
Walking Downstairs	82.93%	100%	100%

**Table 2:** Forecast accuracy for Mental State Classification.

Mental State Classification with Rulex			
Algorithm	LLM	KNN	SVM
Relaxed	92.77%	94.78%	96.39%
Neutral	76.02%	83.74%	89.84%
Concentrating	97.19%	100%	99.60%

**Table 3:** Power consumption for Mental State Classification for HP laptop.

Mental State Classification with Rulex Power Consumption		
Station	LLM	SVM
Time taken	0.27 min.	0.22 min.
Energy (70 Watts)	1120 J.	6230 J.

**Table 4:** Power consumption for Mental State Classification with the Raspberry Pi.

Mental State Classification with Rulex Power Consumption		
Station	LLM	SVM
Time taken	3.33 min.	0.98 min.
Energy (4 Watts)	800 J.	356 J.

**Table 5:** Forecast accuracy for Gender Image Classification.

Gender Detection with Rulex		
Algorithm	KNN	SVM
Female	98.72%	95.60%
Male	94.24%	88.29%

## Data Availability

The Human Activity Detection Dataset using Smartphones can be found in the Kaggle online repository through the following address: https://www.kaggle.com/uciml/human-activity-recognition-with-smartphones. The Mental Sate Detection Dataset can be found in the Kaggle online repository through the following address: https://www.kaggle.com/birdy654/eeg-brainwave-dataset-mental-state. The Vehicle Activity Recognition Dataset can be found in the Kaggle online repository through the following address: https://www.kaggle.com/smartilizer/commercial-vehicles-sensor-data-set. The image-based Gender Classification Dataset can be found in the Kaggle online repository through the following address: https://kaggle.com/ashwingupta3012/male-and-female-faces-dataset.
